# Convergent evolutionary shifts in rhodopsin retinal release explain shared opsin repertoires in monotremes and crocodilians

**DOI:** 10.1098/rspb.2023.0530

**Published:** 2023-04-12

**Authors:** Jinqu Guo, Hai Chi, Linghan Zhang, Shengjing Song, Stephen J. Rossiter, Yang Liu

**Affiliations:** ^1^ College of Life Sciences, Shaanxi Normal University, Xi'an 710119, People's Republic of China; ^2^ School of Biological and Behavioural Sciences, Queen Mary, University of London, London E1 4NS, UK

**Keywords:** monotremes, crocodilians, visual pigment, *in vitro* assay, diel activity

## Abstract

The visual ecology of early mammals remains poorly resolved. Studies of ancestral photopigments suggest an ancient transition from nocturnal to more crepuscular conditions. By contrast, the phenotypic shifts following the split of monotremes and therians—which lost their SWS1 and SWS2 opsins, respectively—are less clear. To address this, we obtained new phenotypic data on the photopigments of extant and ancestral monotremes. We then generated functional data for another vertebrate group that shares the same photopigment repertoire as monotremes: the crocodilians. By characterizing resurrected ancient pigments, we show that the ancestral monotreme underwent a dramatic acceleration in its rhodopsin retinal release rate. Moreover, this change was likely mediated by three residue replacements, two of which also arose on the ancestral branch of crocodilians, which exhibit similarly accelerated retinal release. Despite this parallelism in retinal release, we detected minimal to moderate changes in the spectral tuning of cone visual pigments in these groups. Our results imply that ancestral forms of monotremes and crocodilians independently underwent niche expansion to encompass quickly changing light conditions. This scenario—which accords with reported crepuscular activity in extant monotremes—may help account for their loss of the ultraviolet-sensitive SWS1 pigment but retention of the blue-sensitive SWS2.

## 1. Introduction

The visual abilities and associated ecology of early mammals is poorly understood. Five kinds of visual pigments (opsins with a retinal chromophore) have been recognized across vertebrates: of these, rhodopsin (RH1) underlies dim-light vision, and rhodopsin-like (RH2), middle/long wavelength-sensitive (M/LWS) and two short wavelength-sensitive (SWS1 and SWS2) pigments are each involved in bright-light (colour) vision [1]. However, while comparative studies indicate that ancestral vertebrates possessed all five of these visual pigments [2], the first mammals underwent a loss of the RH2 pigment, resulting in a complement of SWS1, SWS2 and M/LWS for colour vision, and the RH1 for dim-light vision [1,3].

A particular gap in our knowledge concerns the evolutionary forces that led to further changes in vision following the split of the Prototheria (Monotremata) and the Theria (Metatheria and Eutheria) [1,3,4]. Curiously, monotremes lost their SWS1 opsin, whereas therians lost their SWS2 opsin [5]. The current lack of understanding regarding the visual ecology of early monotremes and therians largely stems from an absence of phenotypic data from the photopigments of ancestral and living monotremes [6,7], which has precluded inferences of photopigment evolution and associated shifts in visual ability during the early diversification of mammals.

In recent years, important insights into visual phenotypes have been gained from functional assays of photopigments expressed *in vitro*. For example, measurements of the spectral tuning of cone pigments, quantified as the maximum absorption wavelength (*λ*_max_), have revealed lineage-specific variation relating to photopic niche [7–9]. By contrast, rhodopsin shows rather conserved spectral tuning across mammals, with the exception of some whales and seals [10,11], whereas the rate at which the retinal group is released from the opsin after photobleaching (retinal release rate) appears to vary more widely [6,12]. The retinal release rate of rhodopsin is significantly slower (longer half-life) than that of cone pigments, suggesting a critical phenotype for dim-light sensing [13]. Comparisons among rhodopsins indicate that slow retinal release rates are likely to be adaptive to low light levels in nocturnal species [14], whereas fast rates are better suited to environments in which light levels change rapidly, such as in diving species [15,16].

A smaller number of studies have also expressed proteins inferred from ancestral sequence reconstruction in order to characterize the phenotypes of ancient pigments from extinct taxa [11,17–19]. We previously reported an ancient shift in spectral tuning in M/LWS [20], alongside an acceleration in the retinal release in rhodopsin [21], at the origin of mammals. Both of these findings support a niche expansion from a nocturnal lifestyle to one that encompassed crepuscular conditions. By contrast, the SWS1 pigment appears to be functionally conserved during the evolution of the ancestral mammal, consistent with similar findings from ancestral vertebrates (*λ*_max_ ∼ 360 nm) [17]. Yet despite these results, the spectral tuning of SWS2 in early mammals is still largely unknown, although the extant platypus SWS2 appears to be sensitive to blue wavelengths [7].

Intriguingly, monotremes share an identical photopigment complement with one other vertebrate group—the crocodilians (order Crocodilia)—raising the possibility that these two divergent groups have experienced similar evolutionary pressures acting on their vision [22]. Like monotremes, crocodilians also possess RH1, SWS2 and M/LWS, but have lost their RH2 and SWS1 photopigments. Given that related bird and turtle archosaur lineages have retained all five opsins from the amniote ancestor, it has been suggested that crocodilians underwent a nocturnal bottleneck in their evolution [22]. Although several studies have measured spectral sensitivities of some crocodilian rod and cone photoreceptor cells using microspectrophotometry (MSP) [23–25], currently no information exists on the rhodopsin retinal release rates of crocodilians.

To obtain a more comprehensive understanding of the shifts in visual abilities that took place in the early diversification of mammals, here we combine analyses of molecular evolution with phenotypic assays of multiple photopigments in both living and ancestral monotremes, as well as in crocodilians. We consider the possible conditions that resulted in divergent trajectories in vision between monotremes and therians, and assess whether these show parallels with the divergence of crocodilians and birds. In particular, we hypothesize that the origins of monotremes and crocodilians will show similar visual adaptations, including rhodopsin kinetics, as suggested by their identical complement of photoreceptors and broadly similar diel patterns [26,27].

## 2. Materials and methods

### (a) Opsin coding sequences

For *RH1*, we obtained 117 published mammalian and other tetrapod gene sequences from Liu *et al*. [21] with nine new marsupial sequences obtained from the NCBI database (https://www.ncbi.nlm.nih.gov). We also obtained sequences of *SWS2* (30 species) and *M/LWS* (four species) from NCBI (electronic supplementary material, table S1), again selecting data to cover the focal groups. For *RH1* or *SWS2* gene, orthologues were aligned according to codon position in MEGA X [28].

### (b) Ancestral opsin sequence reconstruction and test of convergence

Ancestral RH1 sequences of each group (mammals, monotremes, marsupials and crocodilians) were based on published data [21]. For mammalian groups, we also repeated ancestral reconstruction with newly available sequences (electronic supplementary material, table S1) using the selected LG + I+*Γ* model by ProtTest 3 [29] in Codeml [30]. Sequence reconstruction was performed under a constrained species tree topology compiled from published data [21,31]. When comparing published and new ancestral reconstructions, we observed no differences for monotremes, one difference for marsupials and therians, and two differences for mammals (electronic supplementary material, figure S1). We also performed ancestral reconstruction for the extinct SWS2 pigment in Codeml, based on a JTT + I+*Γ* model (ProtTest 3) under a species tree topology [32,33] (also see supplementary material data and electronic supplementary material, table S1). We checked our ancestral SWS2 sequence under a free-ratio model (electronic supplementary material, figure S2). The inferred SWS2 sequences of monotreme, crocodilian and archosaur ancestors (electronic supplementary material, data) were synthesized for *in vitro* functional assays.

### (c) Phenotypic assays of mammalian and crocodilian visual pigments

To determine evolutionary and associated ecological shifts in visual ability during the divergence of early mammals or crocodilians, we generated new photopigment phenotype data for several key taxa: the echidna (RH1, SWS2 and M/LWS), platypus (RH1 and SWS2), ancestral monotremes (RH1 and SWS2), ancestral marsupials (RH1), estuarine crocodile (RH1, SWS2 and M/LWS), American alligator (RH1), the crocodilian ancestor (RH1 and SWS2) and archosaur ancestor (SWS2). We used an *in vitro* approach due to the practical and ethical challenges of collecting *in vivo* data from wild vertebrates.

The opsin genes (complete coding regions) were ligated in the vector pcDNA3.1 (+) (Invitrogen), with a tag (5′ ACA GAG ACC AGC CAA GTG GCG CCT GCC 3′) for purification added at the 3′ end of coding sequence and a Kozak sequence (5′ CCACC 3′) at 5′ end. A 48 h transfection was conducted for the plasmid in HEK293T cell line using Xfect reagent (Clontech) and collected cells that containing opsins were then incubated with 11-*cis*-retinal for visual pigment regeneration at 4°C. After solubilization with n-dodecyl β-D-maltoside (Macklin), the visual pigment was purified by Rho 1D4 antibody (The University of British Columbia) in an elution buffer (50 mM [pH = 6.6] HEPES, 0.1% n-dodecyl β-D-maltoside, 140 mM NaCl, 3 mM MgCl_2_ and 20% glycerol added for protein stabilization) containing 40 µM epitope (GenScript) following previous procedures [11,15].

We recorded spectral sensitivity (*λ*_max_) of purified visual pigments (rhodopsin, M/LWS and SWS2) in a U-3900 spectrophotometer (Hitachi). For the M/LWS and SWS2 pigments, to compare with reported values [7,20], a further measurement after light bleaching was performed. Then, a dark (pre-bleaching) minus light (post-bleaching) spectrum was calculated (difference spectrum) to obtain *λ*_max_. For rhodopsin, we measured retinal release rates post-bleaching at 30 s intervals (for 2 s durations) in a Cary Eclipse fluorescence spectrophotometer (Agilent) at 20°C. The excitation (295 nm) and emission wavelengths (330 nm) were set with a 2.5 and 10 nm slit, respectively. The retinal release rate half-life (*t*_1/2_) was calculated as ln2/*b*, by fitting the function *y* = *y*_0_ + *a*(1 − e^−*bx*^) as previously described [15,21]. For each RH1 pigment, 3–5 replicate experiments were carried out, and then statistical tests for *t*_1/2_ values were performed. To eliminate systematic differences between methodologies for protein purification, published data measured with different protocols, such as the echidna RH1 [6], were not included in the statistical tests for retinal release half-lives.

### (d) Mutagenesis

To quantify the phenotypic impact of RH1 amino acid replacements during the origin of monotremes [21] on rhodopsin retinal release, we performed site-directed mutagenesis to generate and characterize 14 mutant proteins. Using the ancestral mammal pigment as a starting point, we initially produced 10 single-mutant pigments, each of which corresponded to one of the different replacements inferred to have occurred at the origin of monotremes (P7Q, N8D, V11I, V81F, L84H, F88L, V137I, A169L, I217T and I318L) [21]. From this set of single-mutants, three showed a greater than 30% shift in retinal release half-life, which we then used to generate three double-mutants (V11I and L84H, V11I and F88L, and L84H and F88L) and one triple-mutant (V11I, L84H and F88L). For each mutant, PCR was conducted using the *FastPfu* DNA polymerase (TransGen Biotech), and PCR products were digested by the *Dpn*I restriction enzyme (New England Biolabs). After sequencing verification, the positive plasmid containing the mutation was transfected into cells and the functional phenotype of the expressed mutant pigment was characterized following the procedures described above.

### (e) Selection tests

For the *RH1* gene, we estimated selection pressures (*ω* or *d*_N_/*d*_S_) acting on the monotreme and crocodilian clades by running separate branch, branch-site and clade models. All selection tests were implemented in Codeml [30] and performed on the established species tree. We first fitted a branch (two-ratio) model, in which we specified different selection pressures on the foreground branch (i.e. ancestor of monotremes or crocodilians, termed *ω*_1_) and on the background (the rest species, *ω*_0_). This was then compared to a one-ratio model in which *ω* was identical across the tree. Significance was assessed by a likelihood ratio test [34]. This was repeated for a three-ratio model in which we assigned different *ω* values to each foreground branch at the same time and compared this to the results of the two two-ratio models.

To gain information on specific sites under selection, we applied a branch-site model to identify site(s) under positive selection on the ancestral branches of monotremes and crocodilians, which we compared with a null model in which *ω* = 1 [35]. Finally, we used clade model C to test for differential selection pressures between each focal clade (i.e. monotremes and crocodilians) and its respective background, which could indicate adaptations to different ecological conditions. The model was then compared to the null model M2a_rel [36,37]. As with the branch model, we then repeated the clade model C for three clades, in which we estimated *ω* for each foreground clade alongside the background.

### (f) Comparison with mutations in retinal disease

Previous work has shown a rhodopsin mutation (G51A) associated with the retinal disease retinitis pigmentosa (RP) in humans can lead to shifts of retinal release rates [38]. To examine whether any of the sites identified in our study are also associated with RP, we searched the ClinVar database (https://www.ncbi.nlm.nih.gov/clinvar) for all mutations listed as potentially implicated in RP in humans. We then compared this set to all derived substitutions on the ancestral branch of monotremes, as well as to the specific sites that we identified from our experiments as being important in altering the retinal release rate.

## 3. Results and discussion

**Figure 1.**
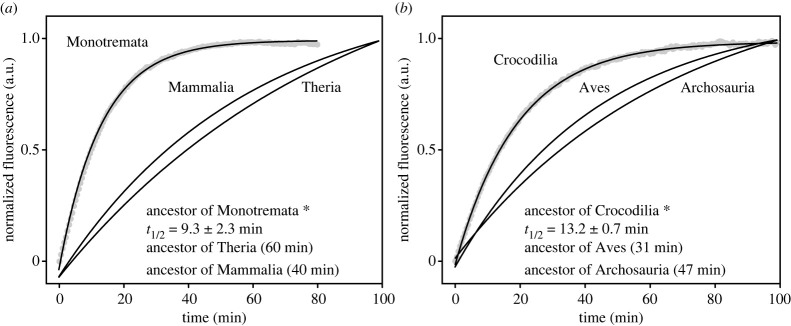
Shift in the rhodopsin retinal release rate at the origins of monotremes and crocodilians. (*a*) Rhodopsin retinal release (arbitrary units, a.u.) of the ancestral monotreme versus the ancestors of Mammalia and Theria. (*b*) Rhodopsin retinal release rate of crocodilian ancestor versus the ancestor of Archosauria. Asterisks denote rates plotted from new measurements (mean and s.d. of *t*_1/2_ values calculated), with others from published data [21].

To determine evolutionary changes in the visual phenotypes of early mammals, we expressed and performed *in vitro* assays of key rhodopsin and cone photopigments from non-placental lineages and compared these to published data from placentals (electronic supplementary material, data). Focusing on the rhodopsin retinal release rate, in contrast with the phenotype of the ancestor of Marsupialia (*t*_1/2_ [half-life of retinal release rate] = 44.9 ± 2.8 min) and also published values of the ancestors of Mammalia (39.9 min), Theria (60.1 min) and Placentalia (54.9 min) [21], we detected a dramatic acceleration in the rhodopsin retinal release rate (*t*_1/2_ = 9.3 ± 2.3 min) at the origin of Monotremata (*p* < 0.001, two-tailed *t*-test for mammalian and monotreme ancestors; figure 1*a*). The rapid rhodopsin kinetics were also seen to be retained by the platypus (9.4 ± 0.8 min) and echidna (12.6 ± 1.7 min) (electronic supplementary material, figure S3a). On the other hand, we detected only minor spectral shifts in the rhodopsin of early mammals (electronic supplementary material, figure S3b).

We compared our results from mammals with data obtained from newly generated rhodopsin proteins from ancestral and extant crocodilians and found striking similarities. Specifically, we observed that rhodopsin retinal release rate was accelerated in the ancestor of Crocodilia (*t*_1/2_ = 13.2 ± 0.7 min) compared to the ancestors of Archosauria (46.6 ± 3.4 min) and birds (30.5 ± 2.3 min) (*p* < 0.001) [21] (figure 1*b*), implying functional convergence with monotremes. This acceleration in retinal release rate was also seen to be retained in the two living crocodilian species examined, the estuarine crocodile (*t*_1/2_ = 13.7 ± 1.8 min) and American alligator (14.1 ± 0.6 min) (electronic supplementary material, figure S3a). As with mammals, we detected negligible shifts in spectral sensitivity (electronic supplementary material, figure S3b).

We tested whether the observed shift in rhodopsin retinal release at the ancestral lineage of monotremes was associated with molecular adaptation and found evidence of a subset of sites (7 and 344, branch-site model) under positive selection in the ancestral branch (electronic supplementary material, table S2). Given that extant monotremes also show rapid release, it appears that the early adaptive phenotypic changes in rhodopsin have been subject to a long period of functional constraint, *ω* = 0.03 (two-ratio and three-ratio models) or 0.08 (clade model C) (electronic supplementary material, table S2), although we cannot rule out phenotypic changes in their extinct relatives. We also found evidence of elevated rates of selection in the *RH1* gene in the ancestral crocodilian (*ω* = 0.16, two-ratio and three-ratio models), and across the clade (*ω* = 0.52, clade model C), although no positively selected sites were detected (electronic supplementary material, table S2).

**Figure 2.**
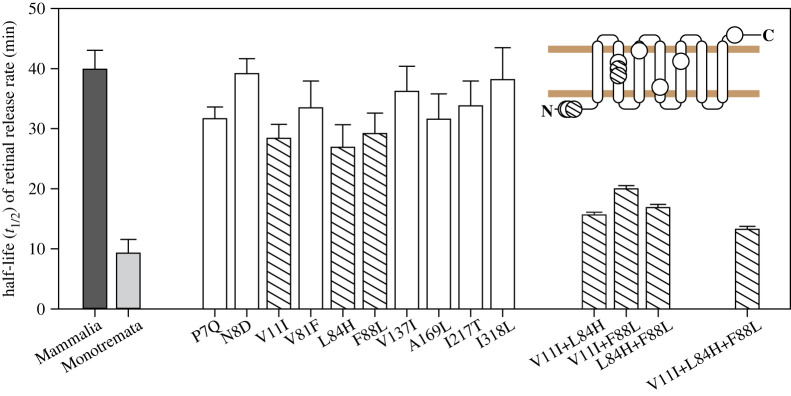
Critical amino acid substitutions underlying the detected shift in retinal release in rhodopsin at the origin of monotremes. Light and dark grey bars indicate newly measured rhodopsin from the monotreme ancestor and published Mammalia value [21], respectively. The three single-mutant pigments that showed the most dramatic shifts of retinal release half-lives, along with three double-mutant and one triple-mutant, are highlighted by hatching. Error bars are standard deviations based on three or four measurements. The 10 amino acid sites are mapped onto a two-dimensional rhodopsin structure [39].

To examine the impact of 10 rhodopsin amino acid replacements that were previously reported to have occurred in the branch leading to monotremes [21], we generated mutant pigments for functional characterization. When compared to the ancestral mammal pigment, we found that five of the 10 mutants (P7Q, V11I, L84H, F88L and A169L) each individually resulted in a significant acceleration (approx. 20–40% shift in half-life) in retinal release rate (*p* < 0.05, one-way ANOVA with *post hoc* Holm-Sidak test), suggesting that the early phenotypic shift involved multiple sites (figure 2; electronic supplementary material, figure S4). We also compared the 10 substitutions to human mutations possibly associated with the disease RP and found that four sites (81, 84, 88 and 137) were common to both sets. Of these sites, two (84 and 88) were among those identified here as being important for retinal release, although the exact replacements were not the same. Remarkably, two of the three most impactful critical substitutions in monotremes (V11I and F88L) were also seen to occur on the ancestral crocodilian branch, raising the possibility that convergent changes in retinal release have arisen via the same mechanism (electronic supplementary material, figure S1).

To test for potential additive or epistatic effects among the residues associated with the greatest shifts in retinal release half-lives, we generated and characterized the phenotypes of double and triple mutants based on the three replacements V11I, L84H and F88L. Measurements from the triple mutant showed that these three amino acid substitutions together accounted for 87% (Δ*t*_1/2_ = 26.6 min) of the phenotypic change from the ancestor of mammals to the ancestor of monotremes (*p* < 0.001, two-tailed *t*-test for the mammalian ancestor and the triple mutant) (figure 2; electronic supplementary material, figure S4), while the double mutant for the two critical sites shared with crocodiles (V11I and F88L) accounted for 65% (Δ*t*_1/2_ = 19.9 min). This raises the possibility that these sites might also contribute to a large proportion of the shift in retinal release half-life between the ancestral archosaur and the origin of Crocodilia.

**Figure 3.**
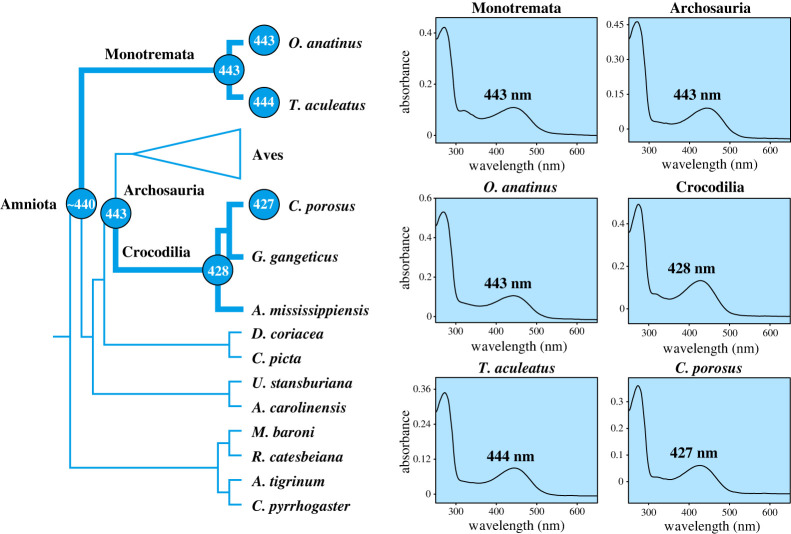
Spectral tuning evolution of the SWS2 pigment since the origins of monotremes and crocodilians (bold lineages). The predicted value for the ancestor of Amniota is indicated by tilde (∼) [40].

We also measured the cone opsin phenotypes for SWS2 for the respective ancestors of monotremes, crocodilians and archosaurs, as well as a representative living taxon from each of these groups. The ancestral monotreme, as well as the platypus and the echidna all showed a maximum spectral sensitivity of 443 to 444 nm. Thus, there appears to have been little change in spectral sensitivity since the amniote ancestor (approx. 440 nm), which is an earlier predicted value [40]. For crocodilians, SWS2 pigments from both the ancestor of Crocodilia and the estuarine crocodile were maximally sensitive at approximately 430 nm, consistent with a shift to shorter wavelengths compared with the archosaur (443 nm) and amniote ancestors (figure 3).

Finally, to obtain information on the M/LWS phenotypes of monotremes and crocodilians, we expressed this pigment based on the echidna and estuarine crocodile. We found that the echidna exhibits only a negligible shift in spectral tuning (*λ*_max_ = 552 nm, electronic supplementary material, figure S5) compared to the published value for the ancestral monotreme [20]. The long period of functional conservation of M/LWS in early monotremes, as well as in therians [20], supports the view that this pigment has remained functionally important throughout mammal evolution. For crocodilians, our *in vitro* assay reveals that the M/LWS pigment of estuarine crocodile has a *λ*_max_ at 543 nm (electronic supplementary material, figure S5). Our measured value therefore corresponds closely to predicted values based on reported critical sites [41] for this taxon and the ancestral crocodilian (both 545 nm), but is smaller than the predicted value of the ancestral archosaur (approx. 560 nm) [22]. Thus, our result adds support to the earlier interpretation of a shift to shorter wavelength sensitivity in the early evolution of crocodilians.

**Figure 4.**
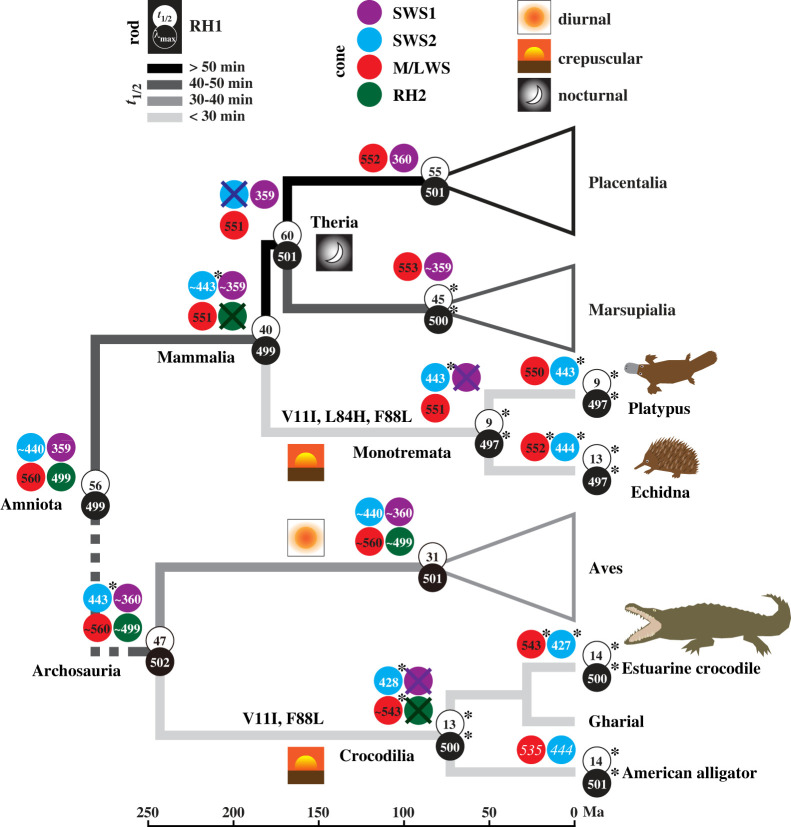
Visual pigment evolution of monotreme and crocodilian ancestors. Branches are shaded from black to pale grey, scaled by faster retinal release rate, that is, smaller *t*_1/2_ values. A dashed line indicates the position of additional lineages (not shown) between the two ancestral nodes. Divergence times for early mammal lineages are based on Upham *et al*. [31]. Two of the three functionally critical sites shared by the ancestors of monotremes and crocodilians are shown. For *λ*_max_, white numbers are dark spectra, black are dark minus light difference spectra. Asterisks denote new measurements and other values are based on published data [7,17,20–22,40,42–44], with predicted values shown as tilde (∼) and two MSP values in italics [24].

Taken together, findings from rhodopsin, SWS2 and M/LWS shed new light on the visual ecologies of early mammals (figure 4). Retinal release rate of rhodopsin is thought to reflect aspects of both diel activity and the photopic environment [14,15,21]. In particular, the faster release rates associated with rapid rhodopsin recharging are likely to be adaptive where vision has to react quickly to fluctuating light levels [45], although direct evidence of this assumption is needed. Following this logic, we propose that acceleration in release rate in ancestral monotremes was likely related to a transition to a crepuscular niche and, specifically, to rapid changes in light levels that occur at dawn and dusk [46,47]. Indeed, hints that the ancestral monotreme was at least partially adapted to non-nocturnal conditions [48,49] also comes from reports that modern monotremes are active at dusk and dawn, or occasionally during the day depending on the season [26,50,51]. Moreover, monotreme rod cells share the same nuclear architecture with many diurnal, but not nocturnal, therian species [52,53].

Similar to monotremes, crocodilians also show crepuscular and even diurnal activity alongside nocturnality [27,54–57]. Therefore, it is plausible that similarities in the rhodopsin phenotype across these two groups represent a case of functional convergence linked to their early visual adaptation to quickly changing twilight niches. For crocodilians, both M/LWS and SWS2 pigments exhibit *λ*_max_-shifts to shorter wavelengths, which are close to published MSP values [23,25].

The addition of new phenotypic data presented in this study helps to explain further the evolution of colour vision in early mammals, and particularly why monotremes lost their SWS1 opsin yet retained their SWS2. Earlier studies have suggested that the ancestral mammal was either adapted to a nocturnal niche, or underwent a shift from a nocturnal niche to one that also included crepuscular conditions [1]. In addition to inferences from RH1 retinal release [21] and M/LWS pigment spectral tuning [20], this scenario is also supported by the loss of the RH2 pigment, which was previously speculated to occur as a result of functional redundancy with RH1 [1,42]. In this context, the inferred transition by ancestral monotremes to twilight conditions would have exposed their retina to potentially more harmful UV wavelengths, and different from some mammals or birds [17], UV light may not have an adaptive role for their vision. Notably, UV vision could be essential for many bird species [58], with several protective mechanisms proposed [59]. Although theoretically an alternative course of evolution might have been for SWS1 to shift to longer wavelengths (violet or blue light) in mammals, as observed in many diurnal therians [9], such a shift in monotremes would have been functionally redundant due to the fact that, unlike therians, they also possessed a blue-sensitive SWS2 (approx. 443 nm). Indeed, the retention of a blue-sensitive SWS2 is consistent with adaptation to a crepuscular niche, given that blue light (approx. 450 nm) is enriched at dusk and dawn [60], which may also be the case for crocodilians.

By comparing the photopigment phenotypes of ancestral and living monotremes and also crocodilians, our results provide the most comprehensive picture to date of the visual evolution of early-stage mammals and archosaurs. More generally, our approach demonstrates the importance of considering the full complement of photopigments for inferring the visual ecology of extinct taxa.

## Data Availability

All data analysed and discussed are provided in electronic the supplementary material [61].
